# Deguelin promotes longevity and healthspan through *C. elegans fmo-4*

**DOI:** 10.17912/micropub.biology.001548

**Published:** 2025-03-11

**Authors:** Angela M Tuckowski, Shijiao Huang, Kelly Chambers, Brandon Buscher, Scott F Leiser

**Affiliations:** 1 Cellular and Molecular Biology, University of Michigan; 2 Biochemistry and Molecular Biophysics, Kansas State University; 3 Molecular and Integrative Physiology, University of Michigan; 4 Pharmacology, University of Michigan

## Abstract

There are multiple approaches to longevity interventions in *Caenorhabditis elegans ,* including genetic factors that are necessary or sufficient for lifespan extension and pharmacological agents that modify physiology to extend lifespan. Many pharmacological interventions act through known genetic pathways to promote longevity. Here, we show that the mitochondrial complex I inhibitor, deguelin, promotes lifespan extension and healthspan in an *fmo-4-* dependent manner. Our results confirm that deguelin increases lifespan and indicate that deguelin induces and requires multiple FMO enzymes to extend lifespan in *C. elegans* , suggesting these enzymes may promote longevity in a coordinated fashion.

**Figure 1. Deguelin requires fmo-4 to promote lifespan extension and improve healthspan in C. elegans.**
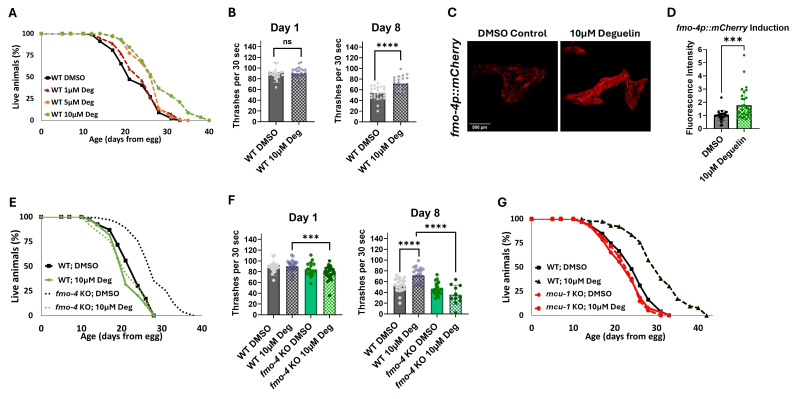
(A) Lifespan assessment of wild-type (WT) worms exposed to DMSO control, 1 µM deguelin, 5 µM deguelin, or 10 µM deguelin (n = ~120 worms per condition, three replicate experiments performed. Significance was determined at p < 0.05 using log-rank analysis). (B) Healthspan analysis of wild-type (WT) worms exposed to DMSO control or 10 µM deguelin thrashing in a drop of M9 solution for 30 seconds on days 1 and 8 of adulthood (n = ~10 worms per condition, three replicate experiments). (C) Fluorescence intensity of *fmo-4 p::mCherry* transcriptional reporter worms exposed to DMSO control or 10 µM deguelin (n = ~20 worms per condition, three replicate experiments), quantified in (D). (E) Lifespan assessment of wild-type (WT) and *fmo-4* knockout (KO) worms exposed to DMSO control or 10 µM deguelin (n = ~120 worms per condition, three replicate experiments performed. Significance was determined at p < 0.05 using log-rank analysis and significant interactions between the condition of interest and genotype was determined at p < 0.01 using Cox regression analysis). (F) Healthspan analysis of wild-type (WT) and *fmo-4* knockout (KO) worms exposed to DMSO control or 10 µM deguelin thrashing in a drop of M9 solution for 30 seconds on days 1 and 8 of adulthood (n = ~10 worms per condition, three replicate experiments). (G) Lifespan assessment of wild-type (WT) and *mcu-1* knockout (KO) worms exposed to DMSO control or 10 µM deguelin (n = ~120 worms per condition, three replicate experiments performed. Significance was determined at p < 0.05 using log-rank analysis and significant interactions between condition of interest and genotype was determined at p < 0.01 using Cox regression analysis). For healthspan and imaging experiments, * denotes significant change at p < 0.05 using unpaired two-tailed t test. NS = not significant. All replicate data can be found in the **Source Data** files.

## Description

Aging is a complex biological process that affects all living organisms, leading to a decline in physiological functions and an increased susceptibility to diseases ^1^ . Studying the aging process is crucial because it allows for a better understanding of how to prevent or delay the onset of multiple age-related chronic diseases simultaneously. The nematode *Caenorhabditis elegans* ( *C. elegans* ) has emerged as a powerful model organism for studying aging due to its short lifespan, well-characterized genetics, and conserved aging pathways ^2^ . Recent research has focused on identifying longevity interventions in *C. elegans* , including genetic factors and pharmacological agents that can extend lifespan and improve healthspan, potentially offering insights into interventions that could promote healthy aging in humans.

A gene family of particular interest are the flavin-containing monooxygenase ( *fmo* ) genes. From this family, *fmo-2* and *fmo-4* are both sufficient and necessary for lifespan extension in *C. elegans* ^3,4^ . These genes play crucial roles in altering cellular metabolism, including one carbon metabolism ^5^ and calcium regulation between the endoplasmic reticulum and mitochondria ^4^ , contributing to the longevity phenotype. Pharmacologically, compounds like deguelin, a natural rotenoid isolated from plants of the *Leguminosae* family, have gained attention for their potential to extend lifespan through the manipulation or inhibition of cellular processes ^6-8^ . For instance, deguelin treatment has been successfully tested as an anti-tumor agent in human cells lines, inhibits mitochondrial complex I, inhibits mammalian target of rapamycin complex I (mTORC1), and extends lifespan in wild-type (WT) *C. elegans* ^6-8^ .

Our lab previously demonstrated that these interventions can act in the same genetic pathway to extend lifespan. Specifically, we have shown that deguelin requires *fmo-2* to promote longevity in *C. elegans ,* suggesting that *fmo-2* acts downstream of deguelin-mediated lifespan extension ^6^ . Furthermore, we established that *fmo-2* also requires *fmo-4* for its overexpression to extend lifespan ^4^ . Given these interconnected relationships, here we investigate whether deguelin and *fmo-4* genetically interact in the context of longevity. Interestingly, our recent publication shows that *fmo-4* extends lifespan downstream of the inhibition of mTORC1 ( *rsks-1* RNA interference) and has genetic ties to mitochondrial metabolism ^4^ . Considering these similarities between *fmo-4* and deguelin, we hypothesized that deguelin may extend lifespan and promote healthspan through the induction of *fmo-4* , and we sought to investigate the downstream effectors involved in this process.

To address this hypothesis, we first examined the effects of deguelin on WT *C. elegans* lifespan. Our lab published that deguelin, in addition to multiple other compounds, extends lifespan of WT worms ^6^ , and so we aimed to validate these data using a range of concentrations. Our results confirm that deguelin extends WT lifespan in a dose-dependent manner ( **Figure 1A** ). To further investigate the quality of life during this extended lifespan, we assessed the healthspan of deguelin-treated worms. Our data show that deguelin not only extends lifespan but also promotes healthspan in middle-aged (day 8 of adulthood) WT worms ( **Figure 1B** ), indicating that the compound may improve overall health and functionality during aging.

To explore the molecular mechanisms underlying deguelin's effects, we focused on the potential involvement of *fmo-4* . We recently found that *fmo-4* is required for mTOR pathway gene *rsks-1-* RNAi-mediated longevity, and that it extends lifespan by regulating calcium signaling between the ER and mitochondria ^4^ . This is interesting because deguelin is an inhibitor of both mTORC1 and mitochondrial complex I ^7^ , providing more evidence for a potential interaction between the two longevity interventions. Using a transcriptional *fmo-4 ::mCherry* reporter, we observed that deguelin treatment induces the expression of *fmo-4* in *C. elegans* by ~2-fold. Fluorescent images and quantification reveal this significant increase in *fmo-4* expression in deguelin-treated worms compared to the DMSO-treated control worms ( **Figure 1C- D** ). This induction suggests that *fmo-4* is regulated by deguelin and may play a crucial role in mediating the longevity-promoting effects of deguelin. To confirm the requirement of *fmo-4* in deguelin-mediated lifespan extension, we performed a lifespan assay using *fmo-4* knockout (KO) worms. Our results demonstrate that the lifespan-extending effect of deguelin is abolished in *fmo-4* KO worms ( **Figure 1E** ), indicating that *fmo-4* is indeed necessary for and downstream of deguelin to extend lifespan in *C. elegans* . Similarly, we found that *fmo-4* is also required for deguelin to promote healthspan in day 8 adult worms ( **Figure 1F** ), further supporting the critical role of *fmo-4* in mediating the beneficial effects of deguelin on aging.

To gain insight into the downstream mechanisms of deguelin-mediated longevity, we investigated the involvement of *mcu-1* , a downstream effector of *fmo-4-* mediated longevity ^4,9^ . *mcu-1* is a mitochondrial calcium uniporter that has been implicated in aging, stress response, and calcium regulation ^4,9^ . Since *fmo-4* is required for the longevity and healthspan effect seen with deguelin treatment, and since *mcu-1* acts downstream of *fmo-4 ^4^ ,* we hypothesized that *mcu-1* would also be required for the benefits of deguelin treatment. Our data show that *mcu-1* is required for deguelin-mediated longevity ( **Figure 1G** ), suggesting that the effects of deguelin on lifespan extension may involve modulation of mitochondrial calcium homeostasis through the *fmo-4 / mcu-1* pathway.

While our findings provide compelling evidence for the role of deguelin in promoting longevity and healthspan in *C. elegans* through the induction of *fmo-4* , there are several limitations to consider. First, the exact mechanism by which deguelin induces *fmo-4* expression remains to be elucidated. It is possible that deguelin, *fmo-2 ,* and *fmo-4* are working in the same genetic pathway to promote these health benefits but more work needs to be done to confirm this involvement. Additionally, further research is needed to determine whether the effects of deguelin on lifespan and healthspan are conserved in other organisms, including mammals. Finally, potential off-target effects of deguelin and long-term consequences of its administration should be carefully evaluated in future studies.

In summary, our data demonstrate that deguelin extends lifespan and promotes healthspan in *C. elegans* in an *fmo-4* -dependent manner. We find that deguelin induces the expression of *fmo-4* and that both *fmo-4* and its downstream effector *mcu-1* are required for deguelin-mediated longevity. These findings contribute to our understanding of the molecular mechanisms underlying lifespan extension and highlight the potential of deguelin as a pro-longevity compound. Future directions for this research include investigating the upstream regulators of *fmo-4* induction by deguelin, exploring the conservation of this pathway in higher organisms, and evaluating the potential of deguelin or related compounds as interventions to promote healthy aging in humans.

## Methods

**Strains and Maintenance**

Standard *C. elegans* cultivation procedures were used as previously described ^3^ . Worm strains were maintained on solid nematode growth medium (NGM) using *E. coli* OP50 throughout life. Worms were transferred using a platinum wire. All worm strains were maintained at 20°C.

**Lifespan Assays**

Gravid adult worms were placed on NGM plates seeded with *E. coli* OP50 for three hours. Then the adults were removed and eggs were allowed to hatch and develop to day 1 adulthood at 20°C. Adult worms were transferred to NGM plates containing 25 µg/mL carbenicillin, floxuridine (FUdR), and either dimethylsulfoxide (DMSO), 1 µM deguelin (Sigma, D0817), 5 µM deguelin, or 10 µM deguelin. Additionally, these plates were seeded with 200 µL of paraformaldehyde (PFA) killed *E. coli* OP50 at a concentration of 3x. Approximately 70 worms were transferred to fresh plates on day 1, day 2, day 4, and day 6 of adulthood. Two plates per strain per condition were tested per replicate experiment. Experimental animals were scored every 2-3 days and considered dead when they did not move in response to prodding under a dissection microscope. Worms that crawled off the plate were not considered, but ruptured worms were considered as previously described ^3^ . Three replicates were performed for each lifespan assay.

**Thrashing Assay**

Worms were synchronized by placing 10 gravid adult worms on NGM plates seeded with *E. coli* OP50 and allowing them to lay eggs for 2 hours at 20°C. The gravid adults were removed and the eggs were allowed to hatch and develop at 20°C until larval stage 2 (L2). At this stage, the L2 worms were transferred to NGM plates containing either DMSO or 10 µM deguelin, seeded with paraformaldehyde killed *E. coli* OP50 . On day 1 adulthood, worms were placed in a drop of M9 solution, as previously described. The body bends were counted at maximum rate for 30 seconds. Thrashing was assayed on day 1 and day 8 of adulthood. The worms that were not used for the day 1 assay were transferred to fresh plates containing either DMSO or deguelin two times until they were ready to be assayed. Three replicates were performed. Data were analyzed in GraphPad Prism using unpaired two-tailed t tests with Welch's correction.

***fmo-4* Induction on Deguelin**

Gravid *fmo-4 p::mCherry* transcriptional reporter adult animals were placed on NGM plates seeded with *E. coli* OP50 . After 3 hours, adults were removed and the eggs were allowed to develop at 20°C until they reached larval stage 4 (L4). Then 30 of the L4 worms were transferred to NGM plates containing either DMSO or 10 µM deguelin and seeded with paraformaldehyde (PFA) killed *E. coli* OP50 . The worms were incubated for 24 hours at 20°C. Then ~20 worms per condition were picked off these plates and added to unseeded NGM plates, anesthetized in 0.5 M sodium azide (Sigma), and imaged at 6.3x magnification with the LASx software and Leica scope using the mCherry fluorescence channel. Three replicates were performed. Each worm was measured for fluorescence in ImageJ. Data were analyzed in GraphPad Prism using t tests.

**Statistical Analyses**

Log-rank test was used to derive p-value for lifespan assays using p < 0.05 cut-off threshold compared to DMSO or wild-type controls. Cox regression was also used to assess interactions between genotype and condition for lifespans using p < 0.01 cut-off threshold compared to controls. **Supplemental Data 1** provide the results of the Log-rank test and Cox regression analyses, which were run in RStudio.

## Reagents

**Table:** 

**Strain Name**	**Genotype**	**Source**
WT or N2	WT	CGC
RB562 ( *fmo-4* knockout)	*fmo-4 ( ok294 ) *	CGC
CZ19982 ( *mcu-1* knockout)	*mcu-1 ( ju1154 ) *	CGC
*fmo-4* transcriptional reporter	*fmo-4 p::mCherry *	Suny Bioscience

**Table:** 

**Reagent**	**Source**	**Catalog #**
Deguelin	Sigma	D0817-5MG
DMSO	Fisher Scientific	BP213-100
Sodium Azide	Sigma	S2002-5G

## Data Availability

Description: Cox regression and Log Rank Analyses. Resource Type: Dataset. DOI: https://doi.org/10.22002/az149-fcy24 Description: Fig 1A replicates. Resource Type: Dataset. DOI: https://doi.org/10.22002/0ppy4-tpv53 Description: Fig 1B replicates. Resource Type: Dataset. DOI: https://doi.org/10.22002/dnfa3-93r97 Description: Fig 1C replicates. Resource Type: Dataset. DOI: https://doi.org/10.22002/dkme1-yyn77 Description: Fig 1D replicates. Resource Type: Dataset. DOI: https://doi.org/10.22002/bef2s-9nq40 Description: Fig 1E replicates. Resource Type: Dataset. DOI: https://doi.org/10.22002/z63ny-qgr42 Description: Fig 1F replicates. Resource Type: Dataset. DOI: https://doi.org/10.22002/76y01-5hj92 Description: Fig 1G replicates. Resource Type: Dataset. DOI: https://doi.org/10.22002/7kr6v-ntb25
