# Corrigendum: Deguelin promotes longevity and healthspan through *C. elegans fmo-4*

**DOI:** 10.17912/micropub.biology.002391

**Published:** 2026-08-31

## Description

**Corrigendum: Deguelin promotes longevity and healthspan through *C. elegans fmo-4***

Tuckowski AM, Huang S, Chambers K, Buscher B, Leiser SF. 2025. Deguelin promotes longevity and healthspan through *C. elegans fmo-4* . microPublication Biology. 10.17912/micropub.biology.001548

In the originally published version of this article, the survival curves in Figure 1E were plotted with incorrect line styles and colors. The wild-type deguelin condition, which should appear as a dashed black line, was instead drawn as a solid green line, leaving the plotted conditions mismatched to the legend.

In the corrected Figure 1E, the four conditions are plotted as follows: wild type + DMSO, solid black line; wild type + deguelin, dashed black line; *fmo-4* knockout + DMSO, solid green line; *fmo-4* knockout + deguelin, dashed green line.

The authors have corrected Figure 1E. The error affected only the graphical display of the data and does not alter the underlying results, statistical analyses, or conclusions of the article.

This error has been corrected online as well as in future PDF versions of this article.

